# Health-Related Quality of Life: Longitudinal Analysis From the Time of Breast Biopsy Into the Post-treatment Period

**DOI:** 10.3389/fgwh.2021.608787

**Published:** 2021-08-16

**Authors:** Michael J. Boivin, Alla Sikorskii, Pamela Haan, Stephanie S. Smith, Laura L. Symonds, Ravindra Khattree, Bruno Giordani, Adrian J. Blow, Janet R. Osuch

**Affiliations:** ^1^Department of Psychiatry, Michigan State University, East Lancing, MI, United States; ^2^Department of Neurology & Ophthalmology, Michigan State University, East Lancing, MI, United States; ^3^Department of Psychiatry, University of Michigan, Ann Arbor, MI, United States; ^4^Department of Surgery, Michigan State University, East Lancing, MI, United States; ^5^Neuroscience Program, Michigan State University, East Lancing, MI, United States; ^6^Department of Mathematics and Statistics, Oakland University, Rochester, MI, United States; ^7^Department of Human Development and Family Studies, Michigan State University, East Lancing, MI, United States; ^8^Department of Epidemiology, Michigan State University, East Lancing, MI, United States

**Keywords:** breast cancer, quality of life, neuropsychology, emotional well-being, spiritual well-being

## Abstract

**Background:** The physical, psychological, social, and spiritual quality of life (QoL) may be affected by breast cancer diagnosis and treatment, with mixed findings for psychological quality of life and cognitive ability performance. The present study aimed to evaluate QoL in women over 1 year from biopsy for a breast abnormality.

**Methods:** Self-reported measures of physical, psychological, social, and spiritual QoL were obtained after biopsy results but prior to treatment initiation (baseline), 4 and 12 months later. CogState computerized neuropsychological screening battery also provided an evaluation of psychological QoL. Three groups of women including those with benign biopsy results, those with malignancy treated with chemotherapy, and those with malignancy not treated with chemotherapy were compared at 4 and 12 months after adjusting for baseline to isolate the effects of treatment. Additional covariates included are age, level of education, and income.

**Results:** Benign biopsy results group included 72 women, whereas malignancy was found in 87 women of whom 33 were treated with chemotherapy and 54 without chemotherapy. At the time of diagnosis, women with cancer had worse psychological and social QoL but better spiritual QoL than those with benign biopsy results. Only CogState monitoring accuracy was worse for women with cancer compared with the controls at the time of biopsy results. After adjusting for QoL at baseline, women treated for cancer had worse physical and social QoL at 4 and 12 months later. Psychological well-being was worse for women with cancer at 4th month but improved at 1 year. No differences in cognition were found at 4 and 12 months when adjusted for baseline cognition and covariates.

**Discussion:** Breast cancer is a traumatic life event for women, affecting psychological and social QoL domains, yet increasing spiritual QoL. Later, cancer treatment worsens physical, psychological, and social QoL compared with those without cancer.

**Conclusions:** These findings suggest that interventions to improve psychological QoL may be especially important at the time of cancer diagnosis, while interventions to improve physical well-being are the most needed during and following cancer treatment. Support to improve social QoL is needed from the time of diagnosis into post-treatment survivorship.

## Introduction

Health-related quality of life (QoL) is a multi-dimensional concept divided into four domains, namely, physical, psychological, social, and spiritual (1, 2). Physical well-being captures physical functional ability, physical symptoms (e.g., pain, nausea), fertility, strength, and sleep. Psychological well-being includes symptoms of anxiety and depression, fear of recurrence, cognition, distress, enjoyment of life, and overall QoL perception. Social well-being reflects family distress, roles, and relationships, appearance, employment, isolation, and finances. Spiritual well-being captures the meaning of illness, religiosity, transcendence, hope, uncertainty, and positive changes. These domains are interrelated with each influencing the other while being affected by health conditions such as cancer and its treatment (3–5).

Breast cancer was the second leading cause of death among women in the United States until 2020 (6) and is expected to surpass heart disease in the next decade (7). A breast abnormality such as a lump begins a cycle of concern around biopsy and its results (8) even if the results indicate no malignancy (9). If malignancy is found, conventional medical care begins with surgery; however, some women may first undergo a course of chemotherapy to shrink the tumor before surgery (neoadjuvant therapy). For early-stage breast cancer, the most common surgery is the breast-conserving lumpectomy with sentinel node mapping. Following surgery, additional symptoms and concerns may include altered body image, pain, decreased arm range of motion, scars, brachial plexopathy, as well as, the knowledge of more advanced cancer with positive nodes. If adjuvant chemotherapy or targeted therapy is indicated, it is administered after surgery, may last for 6–12 months, and cause multiple symptoms, such as fatigue, nausea, and symptoms related to chemotherapy-induced menopause. Radiation therapy is administered after surgery either before or most often after adjuvant chemotherapy. If chemotherapy is not indicated, then 6 week radiation therapy follows surgery. Finally, hormonal therapy or trastuzumab is given for receptor-positive breast cancer for years after chemotherapy and/or radiation is complete (10–12).

For women with breast cancer, the evidence suggests that physical QoL is most affected by chemotherapy as opposed to other treatments (13, 14). More than 60% of women with breast cancer had clinically significant problems with fatigue and sleep (15). The poly-symptom experience of people with solid tumor cancers has been well-documented in the literature (16–19). Among people who have just finished chemotherapy, the median severity of pain and fatigue was 6 on a 0–10 scale (20), and sustained levels of post-treatment pain and fatigue, and peripheral neuropathy have been reported for people who have finished treatment for breast or other solid tumor cancers (21, 22).

Social support is of paramount importance during cancer treatment because it has been shown to reduce the risk of psychological distress and accompanying emotional support has been associated with better social and emotional well-being (23–29). The benefits of social and emotional support for the QoL of a cancer survivor are at least partly related to the reduction in negative appraisal and stressful response to illness (30–35). The spiritual domain of QoL has also been found to be positively related to psychological well-being and negatively related to symptom distress among breast cancer survivors (36, 37).

Psychological distress (depression and anxiety) is highly prevalent in breast cancer survivors and has negative consequences for multiple QoL domains (38–40). Depression occurs in up to 60% of cancer survivors across cancer sites and treatments (41). Between 35 and 65% report anxiety during treatment (42) and 45% experience both depression and anxiety (43, 44). Major depression occurs in approximately 16% of survivors, with subthreshold depressive disorders appearing in almost 22% during treatment (15). These prevalence rates are about 3 times higher than in the general population (45, 46). Psychological distress can interfere with cognitive functioning (e.g., memory, attention, and decision making) (47, 48) and motivation to enact self-management behaviors related to chronic diseases and their treatment (49–51).

The literature on cancer- and treatment-related changes in neurocognitive outcomes has mixed findings. Several studies, meta-analyses, and systematic reviews indicate small to moderate effect sizes for diminished cognitive functioning due to adjuvant chemotherapy (52–54). Another meta-analysis found consistent differences with normative scores only for visual memory, and small effect sizes in comparison to normative scores for executive function, information processing speed, and verbal memory (55). The most recent systematic review and meta-analysis supported elevated risk of neurocognitive dysfunction among women with breast cancer compared with controls, and women treated with chemotherapy compared with other modalities, although, strong statistical evidence was found in only three of 24 studies included in the meta-analysis (56). Mechanisms theorized to underpin cognitive changes have been proposed and included links between cytokines and epigenetic reprogramming (57), particularly with chemotherapy (58, 59). Boivin et al. (60) proposed that immunologic marker CD8+ was sensitive to a broad range of poorer QoL and neurocognitive functioning outcomes, especially in women undergoing chemotherapy (60). Finally, studies of neurocognitive functioning included various measures, from objective performance-based measures to self-report, which are often only weakly correlated and reflect potentially different constructs of cognitive ability (objective tests) vs. the functional effect of cognitive ability in everyday life (self-report) (61).

Understanding which QoL domains affected the most and when, beginning with time from cancer diagnosis and extending past the end of cancer treatment would inform supportive care interventions. This study was a case/control prospective observational cohort comparison (patients with breast cancer biopsy vs. matched control women with a benign biopsy diagnosis). As such, the present study aimed to compare the QoL outcomes in physical, psychological, social, and spiritual domains immediately after biopsy results and 4 and 12 months later among three groups of women with (a) benign biopsy results, (b) malignant tumors not treated with chemotherapy, and (c) malignant tumors treated with chemotherapy. We hypothesized that immediately after biopsy results, the groups with malignancy would have worse psychological QoL, including depression, anxiety, and cognition but better spiritual well-being; at 4 and 12 months, women with malignancy treated with chemotherapy will have worse physical, social, and psychological QoL compared with the other two groups.

## Methods

This research is based on longitudinal data on the QoL for a cohort of women recruited at the time of biopsy for a breast abnormality and followed for 1 year. The study was approved by the Institutional Review Board of Michigan State University and the affiliated oncology settings.

### Sample

The sample included women who underwent a biopsy for a breast abnormality. Following a biopsy, a registered nurse recruiter or a radiological technologist who assisted with interventional breast biopsies approached potential participants for verbal consent to be contacted to learn more about the study. After obtaining the verbal consent, the name of the woman and telephone numbers were given to the nurse who was the project manager. The nurse called to the potential participant to explain the study, and if the woman agreed, informal consent was obtained. Following receipt of written consent and biopsy results, the project manager verified the inclusion and exclusion criteria.

All women had to be 18 years or older, able to speak and read English, and if subsequently diagnosed with breast cancer, treated with breast preservation therapy consisting of lumpectomy and whole-breast irradiation therapy. Exclusion criteria included the diagnosis of any cancer type within the last 5 years (excluding non-melanoma skin cancer), previous diagnosis of breast cancer, diagnosis of a major psychiatric disorder (e.g., bipolar disease and schizophrenia), deafness or blindness, and pregnancy at the time of recruitment. Further, exclusion criteria for the cancer cases included treatment with a mastectomy, involved margins histologically (indication of cancer spread beyond the identified abnormality), lack of planned breast irradiation at the tumor site, the start of systemic therapy (cancer therapy targeting entire body such as chemotherapy or hormonal therapy) prior to the first assessment, and receiving neo-adjuvant therapy (systemic therapy prior to breast cancer surgery).

Exclusion criteria for the control group included any non-solid breast mass, current biopsy results of atypical ductal or lobular hyperplasia or lobular carcinoma *in situ* (indication of no cancer but increased risk of developing cancer), history of lobular carcinoma *in situ* within the last 5 years, fine-needle aspiration biopsy as the only method of diagnosis, or any lesion not arising from the epithelial cells of the breast. Lobular carcinoma is not cancer, but the presence of those cells indicates an increased risk of cancer. Therefore, those women were excluded from the breast cancer group. Consenting women with benign biopsies were put into the control pool. As each cancer case was enrolled, a benign biopsy control was sought using the following probabilistic matching categories such as age (<40 years old, 40–59, and >60), education (some high school/high school diploma and some college/college degree), race (Caucasian and other), and menopause status (premenopausal and post-menopausal), so that the two groups would be demographically comparable to the extent possible. Women in the control pool not matched with any cancer cases within 4 months of consenting were removed from the pool. The first assessment (baseline) was conducted after biopsy results were known (7–10 days after biopsy), and before cancer treatment was initiated for those with malignant biopsy results, except when biopsy also served as surgery to remove the breast abnormality or tumor.

### Enrollment

Of women with a breast cancer diagnosis approached for study enrollment, 87 were enrolled and underwent baseline assessment on the measures described below. Of these participants, two women were excluded: one due to death and the other lost to follow-up when unavailable for further contact, so 85 patients with breast cancer completed all the assessments for this study. The patients with breast cancer approached but were not enrolled, upon screening 139 were ineligible due to one of the exclusion criteria and 105 were eligible for enrollment but declined participation. This resulted in a total of 321 patients with breast cancer being approached for enrollment at the time of diagnosis, 87 of whom were enrolled and 244 who were not.

Seventy-two women with a benign (non-cancer) diagnosis for their breast abnormality biopsy agreed to participate and matched to one of our breast-cancer cases, so they were enrolled and completed the baseline assessment. Five of these women were lost to follow-up (two died, two moved away, and one we lost contact) so that 67 benign biopsy participants completed all the assessments. For the remaining women approached for study participation following benign breast biopsy, 153 agreed to participate and were entered into a pool where they could be matched to a breast cancer participant. However, after 16 weeks these women were removed out from the eligibility list for matching with patients with breast cancer and were not enrolled in the study. Another 313 benign biopsy women declined to participate and 194 were deemed ineligible upon screening (e.g., previous diagnosis of breast cancer or medical history of prior breast abnormalities; self-reported diagnosis of clinical depression, anxiety disorder, or other psychiatric disorder).

### Measures

Questionnaires and tests described below were administered on a laptop computer during clinic appointments at baseline (following the results of the biopsy but before cancer treatment initiation for the women with breast cancer; at the next clinic appointment following biopsy for the benign biopsy participant), as well as 4 and 12 months later. Demographic characteristics including age, level of education, and income were collected at baseline only. The medical information included data on cancer and planned treatments. The second time point (4 months) corresponded to current or recently completed treatment in the chemotherapy group and completed radiation therapy in the non-chemotherapy malignant group, while at the third time point (12 months), cytotoxic chemotherapy was finished, although, hormonal therapy continued to be administered if prescribed. The measures covered the physical, psychological, social, and spiritual domains of the City of Hope Research (HR) QoL. Items referring to cancer were modified to refer to a breast abnormality for the benign control group.

Breast cancer-specific QoL was measured with the instrument developed by researchers at the City of Hope National Medical Center (HR QoL questionnaire). Evidence of validity and reliability has been reported (2, 62). Forty-six items are rated on a 0–10 rating scale and form 5 subscales: physical, psychological, fear, social, and spiritual. Higher scores reflect better QoL. Physical well-being subscale consists of 8 items evaluating fatigue, appetite changes, pain, sleep, weight gain, vaginal dryness/menopausal symptoms, menstrual changes or fertility, and overall physical health. The psychological well-being subscale includes 17 items evaluating coping, happiness, control of life situations, concentration, appearance, single items on depression and anxiety, and items on distress from cancer diagnosis and treatment. Five-item fear subscale queried women on the extent of fear related to a future diagnostic test, second cancer, cancer recurrence, metastasis, and the degree that life is back to normal. The nine-item social well-being subscale evaluates concerns about the family, support from others, relationships, sexuality, employment, activities at home, isolation, financial burden, and concern about breast cancer in female relatives. The spiritual well-being subscale has seven items asking about religious activities, spiritual activities, changes in spiritual life and positive changes in life because of cancer, uncertainty about the future, sense of purpose/mission, and hope. Cronbach's alpha values for five subscales ranged from 0.70 to 0.90 in this study. This tool provided two measures of psychological QoL (psychological well-being and fear subscales), and one measure of each of the physical, social, and spiritual QoL. Additional in-depth measures reflecting the psychological QoL domain were administered to assess depression, anxiety, and cognition.

Depression was measured with the Patient Health Questionnaire (PHQ-9). The PHQ-9 has been reported to have good construct validity and reliability as a depression scale in the general population (63), recognizing both major depression and subthreshold depressive disorder (64). The PHQ brief scale consists of 9 items (PHQ-9) ranked from 0 to 3 and assesses the level of depressed mood over the previous 2 weeks. Total scores for the 9 items range from 0 to 27; the higher the score, the greater the degree of depression. Cronbach's alpha value was 0.84 at baseline.

Anxiety was measured using a short form of the State-Trait Anxiety Inventory (STAI) (65) state anxiety subscale scales (66). This measure consists of 5 items reflecting the state of (situational) anxiety. Trait anxiety measure was not administered because the focus of the study was on the effect of breast cancer diagnosis and treatment (situations) as opposed to a trait of personality. Participants ranked each item on a scale from 1 to 4. Total scores range between 5 and 20; the higher the score, the greater the degree of situational or state anxiety. Cronbach's alpha value was 0.85 at baseline.

CogState is a computerized cognitive assessment battery that uses stimuli consisting of common playing cards within a game-like context, making the assessment both less stressful and more engaging. Tests from CogState selected for this study assessed a wide range of cognitive ability domains including simple (Detection, DET) and choice (Identification, IDN) reaction time for attention, working memory (One Card Back, OCB), episodic learning, and memory (One Card Learning, OCL, and Continuous Paired-Associate Learning, CPAL), dual-task performance (Monitoring MON), and reasoning (Prediction, PRED). The entire battery took approximately 15–20 min to complete. Within Cogstate, equivalent stimuli are randomly chosen for each response trial, so repeated assessments can take place with minimum confounding from practice effects. CogState has good sensitivity and specificity in classifying mild cognitive impairment and has been shown the ability to detect the cognitive change in response to disease or its treatment (67–69). At the end of the CogState performance, items are a set of short questions where the respondent evaluates her performance on the test in terms of alertness, vigilance, memory, processing speed, and monitoring. In the present analyses, these responses are referred to as subjective metacognition self-ratings.

### Statistical Analyses

Descriptive statistics were obtained for each group such as those with benign biopsy results; those with malignant tumors not treated with chemotherapy; and those with malignant tumors treated with chemotherapy. The chi-square and ANOVA were used to compare the groups at baseline (87 breast cancer and 67 benign biopsy women). Linear mixed-effects (LME) models were employed to relate outcomes at 4 and 12 months and to relate outcomes at intake and covariates (age, level of education, and income). The LME modeling generalizes classical analysis of repeated measures and allows for data missing at random and structured covariance matrix. Even though, only 67 of the 72 benign biopsies enrolled women and 85 of the 87 enrolled women with breast cancer completed all the assessments, they could all still be included in the LME modeling analysis for the repeated measures because of how it allows for missing data. We also repeated the LME modeling analyses as shown in **Table 2** including only the 67 benign biopsies and 85 women with breast cancer who completed all the assessments to see if any of the significant statistical findings would change however they did not so enrolled women were included in the LME modeling as shown in **Table 2** using all available assessment data.

The essential parameters of interest in these models were associated with the group-by-time interaction. The least-square (LS) means for each group at 4 and 12 months were output from the LME models, and differences among LS means were tested. For the hypothesis about the physical QoL, City of Hope physical well-being scores were analyzed. For the comparison of psychological QoL, City of Hope psychological well-being and fear, and PHQ-9, STAI, and CogState were analyzed. For the comparison of social and spiritual QoL, the corresponding subscales of the City of Hope QoL tool were used. Because all outcomes were defined *a priori*, no adjustments for multiple testing were made.

### Sample Size and Power Considerations

The sample sizes for each group could not be set up *a priori* as women were enrolled prior to biopsy results and determination of treatment plan. Given the available sample sizes of 72, 54, and 33, the effect sizes (Cohen's *d*) detectable as statistically significant with the power of 0.80 in two-tailed tests at 0.05 level of significance in unadjusted pairwise group comparison were *d* = 0.51 and 0.59 for the comparison of the benign group to two cancer groups, and *d* = 0.63 for the comparison between two cancer groups. In longitudinal analyses of two repeated measures with the adjustment for baseline, with a correlation of 0.4 between pairs of repeated measures, the detectable adjusted effect sizes were *d* = 0.47, 0.55, and 0.58, respectively.

## Results

The entire sample of 159 women included 87 women with histologically proven breast cancer defined as ductal carcinoma *in situ* or invasive ductal or invasive lobular carcinoma and 72 women with a non-proliferative epithelial cell breast biopsy performed within the previous 4 months. The demographic characteristics of the study sample and descriptive statistics of the outcomes at baseline for the entire sample and three groups of interest are summarized in Table 1. A total of five women (three in benign group, one in chemotherapy, and one in no chemotherapy group) dropped out between intake at 4 months, and an additional two women in the benign group were lost to follow-up between 4 and 12 months. Women with malignant biopsy results not treated with chemotherapy were older, whereas those treated with chemotherapy had worse psychological and social QoL at baseline, and worse scores on depression at baseline. The benign group had the lowest spirituality scores at baseline. Among CogState scores, only monitoring accuracy was worse in the group that later received chemotherapy (Table 1).

**Table 1:** Demographic characteristics and outcomes at intake.

**Characteristic**	**Entire sample *N* = 159**	**Benign group *N* = 72**	**No Chemotherapy group *N* = 54**	**Chemotherapy group *N* = 33**	***P*-value for group comparison**
	***N* (%)**	***N* (%)**	***N* (%)**	***N* (%)**	
Education					0.34
High school or less	40 (25.16%)	15 (20.83%)	17 (31.48%)	8 (24.24%)	
Some college	62 (38.99%)	29 (40.28%)	17 (31.48%)	16 (48.48%)	
College degree	23 (14.47%)	11 (15.28%)	6 (11.11%)	6 (18.18%)	
Post-graduate work or degree	34 (21.38%)	17 (23.61%)	14 (25.93%)	3 (9.09%)	
Income					0.50
< $30,000	46 (28.93%)	23 (31.94%)	11 (20.37%)	12 (36.36%)	
$30,000–$75,000	54 (33.97%)	24 (33.33%)	21 (38.89%)	9 (27.27%)	
More than $75,000	59 (37.11%)	25 (34.72%)	22 (40.74%)	12 (36.36%)	
	Mean (St Dev)	Mean (St Dev)	Mean (St Dev)	Mean (St Dev)	
Age	55.70 (8.72)	54.57 (8.61)	58.80 (8.32)	53.09 (8.41)	**<0.01**
Physical QoL^*^	65.70 (12.54)	67.53 (12.47)	65.99 (10.93)	61.20 (14.31)	0.05
Psychological QoL^*^	103.54 (18.95)	109.25 (15.03)	101.56 (19.49)	94.32 (21.79)	**<0.01**
Fear^*^	24.21 (11.82)	26.27 (11.15)	23.57 (11.88)	20.76 (12.56)	0.37
Social QoL^*^	70.37 (14.06)	76.92 (10.46)	68.37 (13.33)	59.36 (14.51)	**<0.01**
Spiritual QoL^*^	48.19 (13.25)	44.76 (12.99)	50.53 (12.17)	51.84 (14.06)	**0.01**
Depression^**^	4.87 (4.79)	4.03 (4.50)	4.74 (4.48)	6.92 (5.40)	**0.02**
Anxiety^**^	7.68 (2.93)	7.54 (2.91)	7.80 (2.88)	7.79 (3.10)	0.87
Metacognitive score^*^	21.13 (4.56)	20.68 (4.28)	21.07 (5.34)	22.18 (3.63)	0.30
CogState identification time^**^	2.78 (0.10)	2.76 (0.09)	2.79 (0.11)	2.79 (0.10)	0.24
CogState detection time^**^	2.55 (0.12)	2.54 (0.12)	2.56 (0.12)	2.56 (0.12)	0.47
CogState associate learning accuracy^*^	0.84 (0.16)	0.86 (0.14)	0.85 (0.15)	0.79 (0.21)	0.08
CogState spatial learning/working memory accuracy^*^	0.53 (0.27)	0.59 (0.31)	0.50 (0.23)	0.47 (0.22)	0.06
CogState monitoring accuracy^*^	1.10 (0.21)	1.15 (0.19)	1.11 (0.20)	0.97 (0.23)	**<0.01**
CogState non-verbal learning/memory accuracy (one card learning)^*^	0.80 (0.13)	0.81 (0.13)	0.78 (0.11)	0.79 (0.17)	0.48
CogState working memory accuracy (one card back)^*^	1.31 (0.23)	1.34 (0.24)	1.31 (0.19)	1.24 (0.25)	0.11
CogState reasoning/problem solving accuracy (prediction accuracy)^*^	0.88 (0.12)	0.90 (0.13)	0.88 (0.12)	0.86 (0.11)	0.16

* *Higher score indicates better outcome*.

** *Higher score indicates worse outcome*.

*Statistically significant probability (P) values for between-group differences are in bold*.

### An Unadjusted Comparison of Study Groups at 4 Months Post-diagnosis on QoL

**Figure 1:**
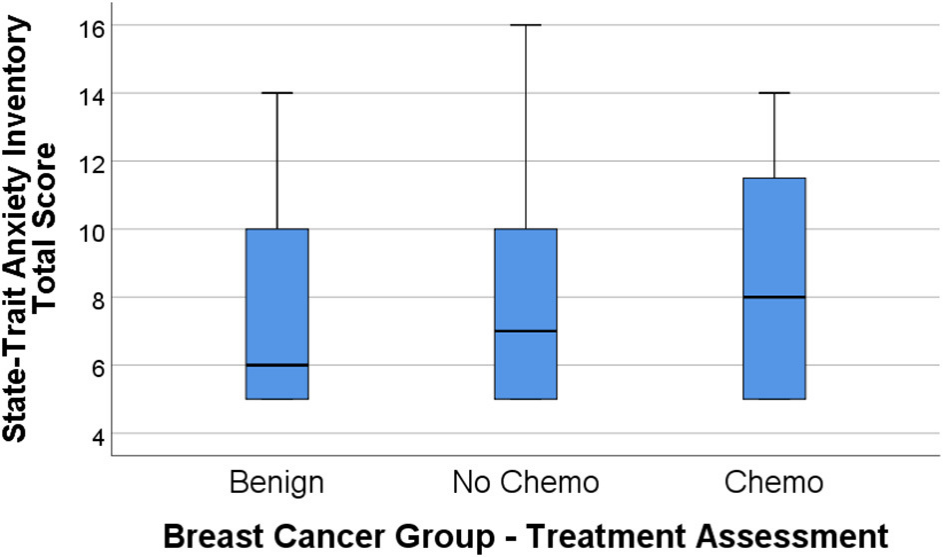
State-Trait Anxiety Inventory (STAI) total score box plots for the benign (breast lump autopsy), no-chemo (treatment for breast cancer), and chemo (treatment for breast cancer) groups. Higher scores mean greater total anxiety, with the median group value-line bisecting the box, top and bottom of the box representing 3rd and 1st percentile, and upper-value range of scores capped above the box.

**Figure 2:**
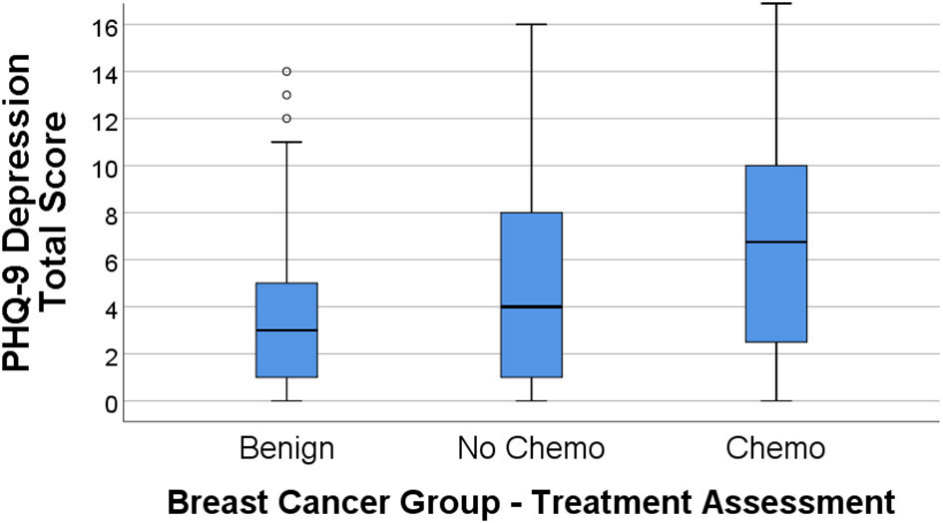
PHQ-9 depression total score box plots for the benign (breast lump autopsy), no-chemo (treatment for breast cancer), and chemo (treatment for breast cancer) groups. Higher scores mean greater total depression, with the median group value-line bisecting the box, top and bottom of the box representing 3rd and 1st percentile, and upper-value range of scores capped above the box (along with outliers for the benign group).

Figures 1, 2 display the box plot comparison of State-Trait Anxiety Inventory (STAI) total and PHQ-9 Depression inventory total for the benign biopsy (no breast cancer) group, no chemotherapy breast cancer, and chemotherapy breast cancer treatment groups at 4 months post-diagnosis (during treatment). The chemotherapy group had more anxiety and depression symptoms (higher median total scores) than the no-chemotherapy breast cancer group. Similarly, the no-chemotherapy group was poorer on these indicators than the benign biopsy (no breast cancer) group.

**Figure 3:**
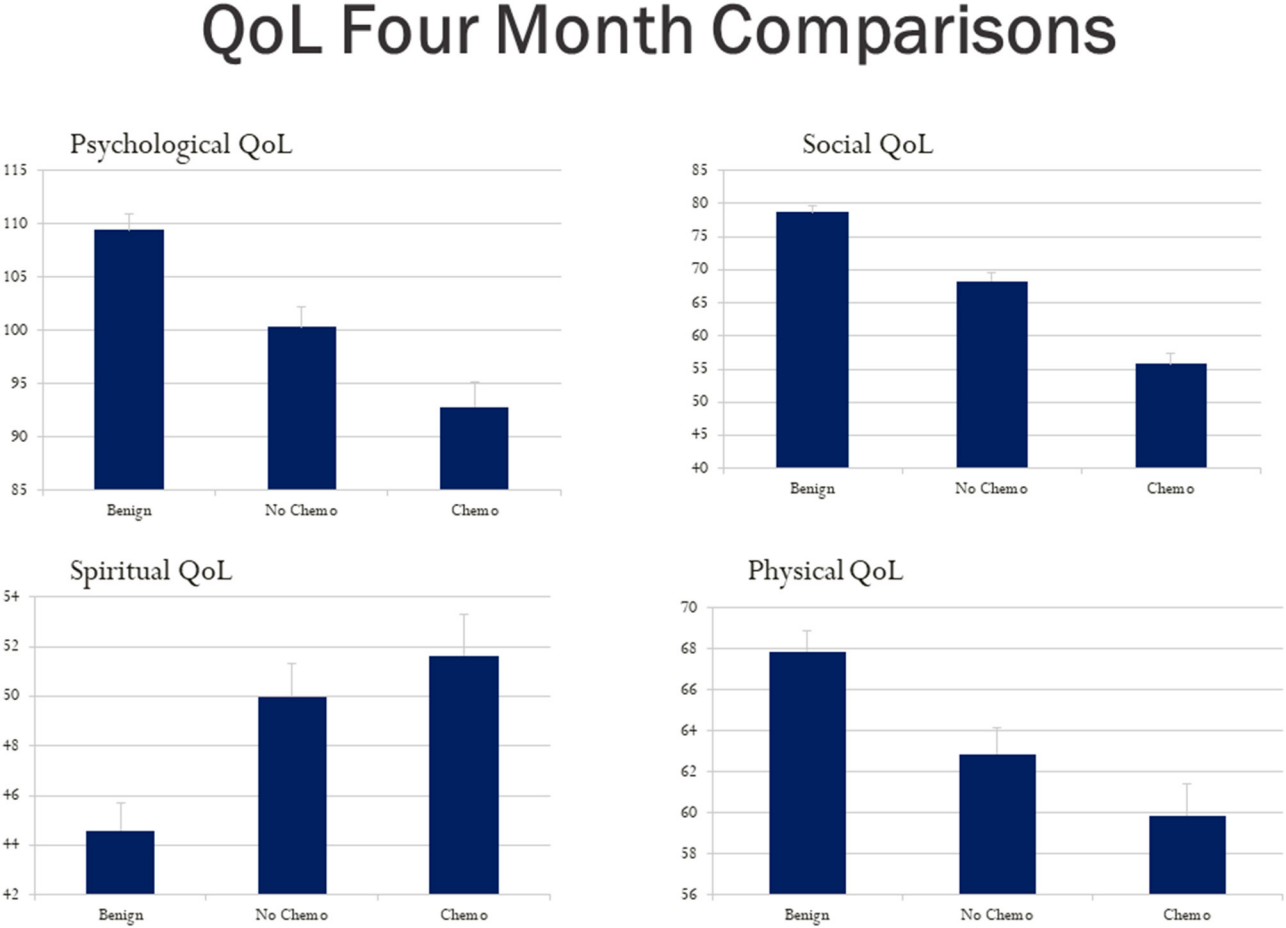
Hope Quality of Life (QoL) questionnaire total score bar graphs for the benign (breast lump autopsy), no-chemo (treatment for breast cancer), and chemo (treatment for breast cancer) groups. Higher scores mean better QoL for the psychological, social, spiritual, and physical domains respectively. The SE value for each group is capped above its bar.

**Figure 4:**
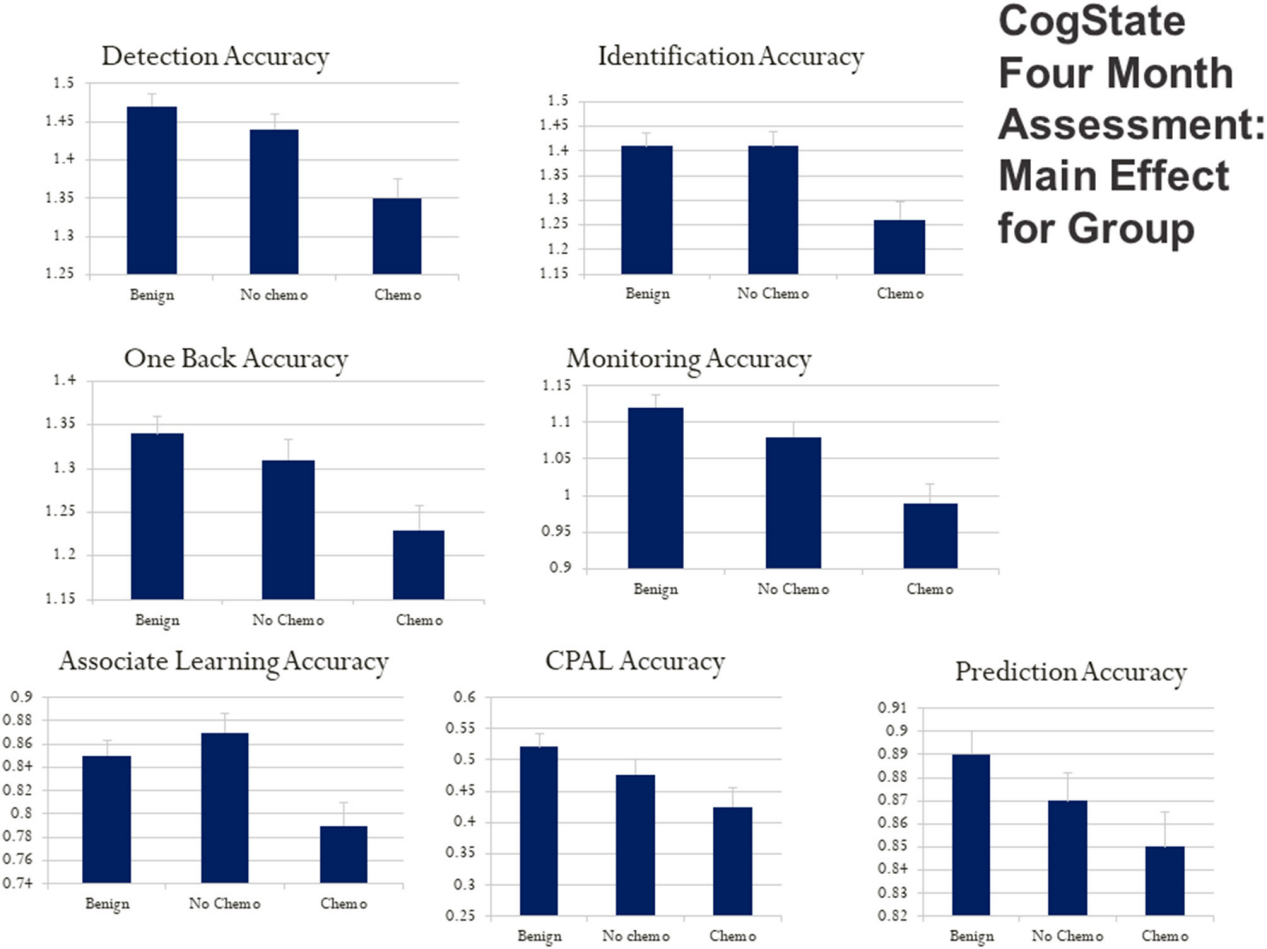
CogState computerized cognitive ability test score box plots for the benign (breast lump autopsy), no-chemo (treatment for breast cancer), and chemo (treatment for breast cancer) groups. Higher scores mean better cognitive performance on the battery of tests with playing cards. Box plots are presented for card detection accuracy, card identification accuracy, one-back card memory accuracy, card monitoring (prior card recognition learning) accuracy, card paired-associate learning accuracy, continuous paired-associate learning accuracy (CPAL), and card presentation pattern prediction accuracy. The SE value for each group is capped above its bar.

Using a bar graph with the SE extension for each group, Figure 3 depicts the unadjusted differences among our study group participants at 4 months post-diagnosis on the City of Hope QoL domains. Figure 4 is a box plot comparison for our comparison groups for cognitive performance tests comprising the computerized CogState neuropsychological cognitive performance screening battery. The chemotherapy treatment group was noticeably worse (lower scores) on all the QoL domains (Figure 3) and the CogState performance tests (Figure 4) compared with the no chemotherapy breast cancer and benign biopsy (no breast cancer) groups.

### Longitudinal Analyses of Outcomes at 4 and 12 Months Adjusted for Baseline

Controlling for baseline scores allowed the longitudinal effects of treatment to be isolated over and above what could be attributed to the impact of initial diagnosis, with further adjustment for age, education, and income. Significant differences in outcomes as time progressed were found in physical, psychological, and social HR QoL as well as depression. The pattern of these differences was the same across multiple outcomes. The group with malignant tumors treated with chemotherapy had worse outcomes compared with the other two groups (Table 2). Notably, over and above baseline values, no differences were found on spirituality, subjective (metacognition), or objective (CogState) measures of cognitive functioning. As for the within-group changes between 4 months and 1 year, the chemotherapy group had significant improvements in physical, psychological, and social well-being, and depression.

**Table 2:** Least square (adjusted) means and SE of outcomes at 4 months and 1 year adjusted for intake, age, education, and income.

**Outcome**	**Benign group**	**No chemotherapy group**	**Chemotherapy group**	***P*-value for group differences at 4 months *P*-value for group differences at 1 year**
	**LS mean (SE)**	**LS mean (SE)**	**LS mean (SE)**	
Physical QoL^*^				
4 months	67.01 (1.22)	61.70 (1.41)	59.05 (1.81)	**<0.01**
1 year	68.29 (1.24)	61.81 (1.41)	67.20 (1.81)^***^	**<0.01**
Psychological QoL^*^				
4 months	106.02 (1.78)	103.50 (2.03)	93.94 (2.61)	**<0.01**
1 year	107.35 (1.80)	106.50 (2.03)	104.08 (2.61)^***^	0.59
Fear^*^				
4 months	26.62 (0.98)	24.86 (1.11)	26.45 (1.43)	0.46
1 year	27.98 (0.99)	24.98 (1.11)	26.12 (1.45)	0.13
Social QoL^*^				
4 months	76.54 (1.22)	71.28 (1.33)	61.62 (1.81)	**<0.01**
1 year	76.87 (1.23)	71.60 (1.33)	69.85 (1.80)^***^	**<0.01**
Spiritual QoL^*^				
4 months	46.74 (1.05)	48.32 (1.20)	47.75 (1.54)	0.61
1 year	49.11 (1.07)	48.21 (1.19)	49.52 (1.54)	0.76
Depression^**^				
4 months	4.36 (0.47)	5.03 (0.55)	5.74 (0.55)	0.25
1 year	4.14 (0.48)	4.57 (0.54)	3.11 (0.70)^***^	0.26
Anxiety^***^				
4 months	7.47 (0.33)	7.79 (0.38)	8.05 (0.49)	0.58
1 year	7.44 (0.34)	7.11 (0.38)	7.71 (0.49)	0.62
**Metacognitive score**				
4 months	22.37 (0.48)	22.57 (0.55)	22.05 (0.70)	0.84
1 year	21.11 (0.48)	22.08 (0.55)	21.83 (0.70)	0.40
CogState identification time^**^				
4 months	2.78 (0.01)	2.77 (0.01)	2.78 (0.01)	0.64
1 year	2.77 (0.01)	2.77 (0.01)	2.79 (0.01)	0.52
CogState detection time^**^				
4 months	2.54 (0.01)	2.57 (0.01)	2.58 (0.02)	0.17
1 year	2.55 (0.01)	2.56 (0.01)	2.55 (0.02)	0.92
CogState associate learning accuracy^*^				
4 months	0.85 (0.02)	0.87 (0.02)	0.83 (0.03)	0.44
1 year	0.84 (0.02)	0.82 (0.02)	0.80 (0.03)	0.44
CogState spatial learning/working memory accuracy^*^				
4 months	0.45 (0.03)	0.43 (0.03)	0.42 (0.04)	0.78
1 year	0.47 (0.03)	0.45 (0.03)	0.44 (0.04)	0.77
CogState monitoring accuracy^*^				
4 months	1.08 (0.02)	1.04 (0.03)	1.08 (0.03)	0.46
1 year	1.09 (0.03)	1.08 (0.03)	1.07 (0.04)	0.85
CogState non-verbal learning/memory accuracy (one card learning)^*^				
4 months	0.82 (0.02)	0.79 (0.02)	0.81 (0.02)	0.51
1 year	0.82 (0.02)	0.77 (0.02)	0.80 (0.03)	0.17
CogState working memory accuracy (one card back)^*^				
4 months	1.33 (0.03)	1.31 (0.03)	1.25 (0.04)	0.38
1 year	1.32 (0.03)	1.29 (0.04)	1.23 (0.05)	0.27
CogState reasoning/problem solving accuracy (prediction accuracy)^*^				
4 months	0.89 (0.01)	0.86 (0.02)	0.86 (0.02)	0.49
1 year	0.89 (0.02)	0.86 (0.02)	0.86 (0.02)	0.55

* *Higher score indicates better outcome*.

** *Higher score indicates worse outcome*.

*** *Significant change over time within group*.

*Statistically significant probability (P) values for between-group differences are in bold*.

## Discussion

The comparison of groups with benign vs. malignant findings adds to the existing literature on the effects of breast cancer diagnosis and chemotherapy treatment on physical, psychological, social, and spiritual QoL. When comparing the benign biopsy (no breast cancer) group, no chemotherapy breast cancer, and chemotherapy breast cancer treatment groups, differences at the time of diagnosis were found on psychological, social, and spiritual QoL, favoring the benign group except for spirituality. Controlling for the QoL measures at the time of diagnosis and age, education, income, and chemotherapy group had more depressive symptoms than the no chemotherapy breast cancer and the control groups. We observed higher (better) QoL scores for the benign biopsy than the breast cancer groups on psychological, social, and physical QoL domains, respectively; and the chemotherapy treatment group had the worse scores for all these domains. However, the breast cancer groups were higher on spiritual QoL (more affirmation of positive spirituality items) than the benign (no breast cancer) group, even though, they were comparable at diagnosis. The fact that spiritual QoL can be higher for the participants with breast cancer during treatment while psychological and social QoL can be worse (compared with the benign biopsy group) is important to note. This is because this finding suggests that spirituality is not necessarily dependent on emotional well-being during treatment for a life-threatening disease for women diagnosed with breast cancer. Higher spirituality may buffer psychological distress resulting from cancer diagnosis (70).

These findings regarding depressive symptoms (part of psychological QoL) align with the literature indicating that breast cancer survivors have greater mental health problems than cancer-free controls (56, 71–73). The previous qualitative analysis revealed that all women in this sample reported a difficult time pre-diagnosis (74). Because enrollment into the present study happened at the time of biopsy for a breast abnormality, we were able to discern the effect of the diagnosis vs. subsequent treatment. Greater depressive symptoms in the cancer groups were found at the time of diagnosis, but not 4 months and 1 year later when controlling for depression at the time of diagnosis. This finding is consistent with the literature on high (50%) prevalence of depressive symptoms shortly after cancer diagnosis and diminishing over the next year, while remaining higher than in the general population even years after cancer treatment completion (75–77). Even though, for most people with cancer depressive symptoms are not sufficiently severe to warrant a full clinical diagnosis of depression (78–81), but depressive symptomatology needs attention, as it has been suggested to be a predictor of better survival and reduced morbidity in several cancer populations (82).

Further, cancer treatment gives rise to multiple physical and emotional symptoms (19). The biological changes due to chemotherapy or other treatments and the resulting inflammatory processes may be responsible for the impairments in physical QoL (17, 83, 84), and these findings regarding physical QoL agree with the literature and contribute to the evidence that even a year after the cancer diagnosis, physical QoL remains worse for women with cancer compared to controls.

On the other hand, for some of the problems such as memory and concentration, chemotherapy may not be the cause, but rather, the stress of the diagnosis may be a significant contributor to early concerns expressed by women with cancer. The objective computer-based measures of cognition and attention showed no differences over time (85). It is possible that if patients report perceived problems with cognition (sometimes referred to as “chemo brain”), a broad evaluation may be needed to identify contributing issues related to all domains of QoL. The findings of this research are consistent with those obtained by Darby (86) in a study of 60 women (30 on chemotherapy and 30 controls), who found some problems in attention and learning prior to the start of chemotherapy, with only minor changes during and after the chemotherapy period (86). Neither cognitive test scores nor self-report of their cognitive performance of the women differed over the course of treatment or by the group; though initially, at the time of diagnosis before the initiation of treatment, the chemotherapy group demonstrated lower monitoring accuracy compared with the other two groups.

The effects of breast cancer disease and treatment, especially chemotherapy, on neuropsychological functioning have been examined in studies with patients with breast cancer over decades (87–89). The cognitive fatigue from the psychosocial stress associated with breast cancer disease and treatment may be largely responsible for diminished neuropsychological performance in people on active treatment (90) and years after finishing treatment (91). Finally, the use of immunological biomarkers during differing types of breast cancer treatment may help disentangle the effects of the disease itself and its treatment from the psychosocial stress, causing cognitive fatigue and diminished capacity (92, 93).

The limitations of the present study include relatively small sample size and unequal group sizes resulting from biopsy results and planned treatment. Women in the sample were also well-educated and the results may not generalize to those with lower education levels. Because the women in the sample were not randomly assigned to groups receiving and not receiving chemotherapy, any differences found between these groups in the outcome variables must be interpreted cautiously. Further, multiple clinical factors and preferences of women played a role in treatment decisions after a malignant biopsy result. We did not disentangle these factors because data on preferences of the women and medical record data were not uniformly available. Even if clinical data were available, extraneous confounding variables cannot be ruled due to the observational nature of this study.

## Conclusions

These findings and those of others suggest that interventions to improve the emotional and cognitive components of QoL may be especially important at the time of cancer diagnosis. A threatening illness, such as breast cancer, can be conceptualized as a traumatic event in the life of a woman and her significant others. While the benign group experienced relief after diagnosis, the emotional problems intensified among those diagnosed with cancer. The cancer diagnosis experience evokes emotions and memories; and often these are negative, causing distress, which may affect multiple other QoL components. Supportive care interventions initiated at the time of diagnosis may be particularly impactful for psychological QoL, including depression and cognition.

## Data Availability Statement

The raw data supporting the conclusions of this article will be made available by the authors upon request, without undue reservation.

## Ethics Statement

The studies involving human participants were reviewed and approved by Michigan State University's Biomedical & Health Institutional Review Board (BIRB) application #05-1031S Breast Cancer SEQL (Spirituality, Emotional Well-being and Quality of Life) Project (IRRC#6059M) (PI: Michael J. Boivin). The patients/participants provided their written informed consent to participate in this study.

## Author Contributions

All authors listed have made a substantial, direct and intellectual contribution to the work, and approved it for publication.

## Conflict of Interest

The authors declare that the research was conducted in the absence of any commercial or financial relationships that could be construed as a potential conflict of interest.

## Publisher's Note

All claims expressed in this article are solely those of the authors and do not necessarily represent those of their affiliated organizations, or those of the publisher, the editors and the reviewers. Any product that may be evaluated in this article, or claim that may be made by its manufacturer, is not guaranteed or endorsed by the publisher.
